# Supplementary material for: Cross-subject mapping of neural activity with restricted Boltzmann machines
Source: Front Comput Neurosci. 2026 Feb 13;20:1710914. doi: 10.3389/fncom.2026.1710914 (PMC12945994; doi:10.3389/fncom.2026.1710914)
Supplement: Supplementary file 1 [file Data_Sheet_1.pdf]

1 SUPPLEMENT DATA OF CROSS-SUBJECT MAPPING OF NEURAL ACTIVITY WITH RESTRICTED BOLTZMANN MACHINES

This is the extended data of Cross-subject Mapping of Neural Activity with Restricted Boltzmann Machines. In this extended data we include two additional experiments that complement the main text: cross-validation result, comparison with persistent contrastive divergence, and an additional experiment that studies the effect of the number of sampling steps k.

2 COMPARISON WITH PERSISTENT CONTRASTIVE DIVERGENCE

Outside from standard contrastive divergence, Tieleman (2008) proposed persistent contrastive divergence as a improved method for training and RBM. In Figure 9, we empirically show that PCD does improve the decoding accuracy, raising the performance to on par with our proposed Fisher divergence.

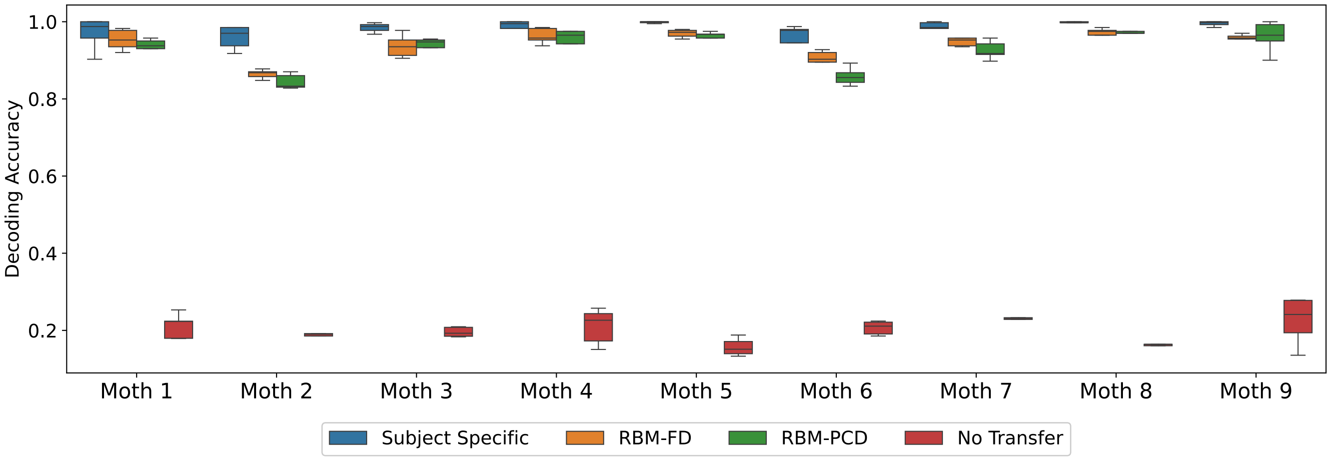

Fig 9. Scenario 1 experiment but repeated with persistent contrastive divergence.

3 ALTERING k DURING SAMPLING

In the main text, we used sampling hyperparameter $k = 1$ to sample target distributions in the source domain. In Figure 1–1 and 1–2, we empirically show that the choice of sampling step k marginally changes the experiment results. In some particular cases, increasing the number of gibbs sampling step could improve result (eg. Moth 4 contrastive divergence), and sometimes decrease performance variance (Moth 3, 4 fisher divergence for k=10), but these improvements are not consistent for all moths. These empirical results lead to our conclusion that the effect of k in model performance is minor and for better efficiency we use $k = 1$.

4 CROSS VALIDATION

In this section, we discuss the 5-fold cross validation result. We rerun the empirical experiment using 5-fold cross validation where 80% of the data is separated as the training set and the rest of 20% is used as the validation set. The training and validation process is then repeated 5 times in a rolling basis such that all part of the data is used as validation once. The cross validation results for the two scenarios are presented

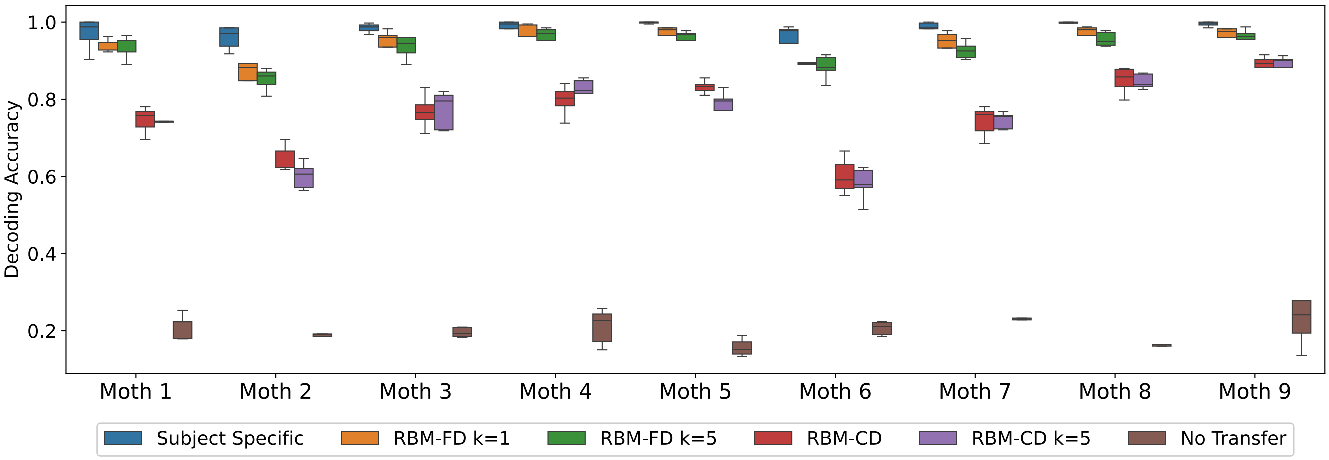

Fig 1–1. Scenario 1 experiment but repeated with k=5.

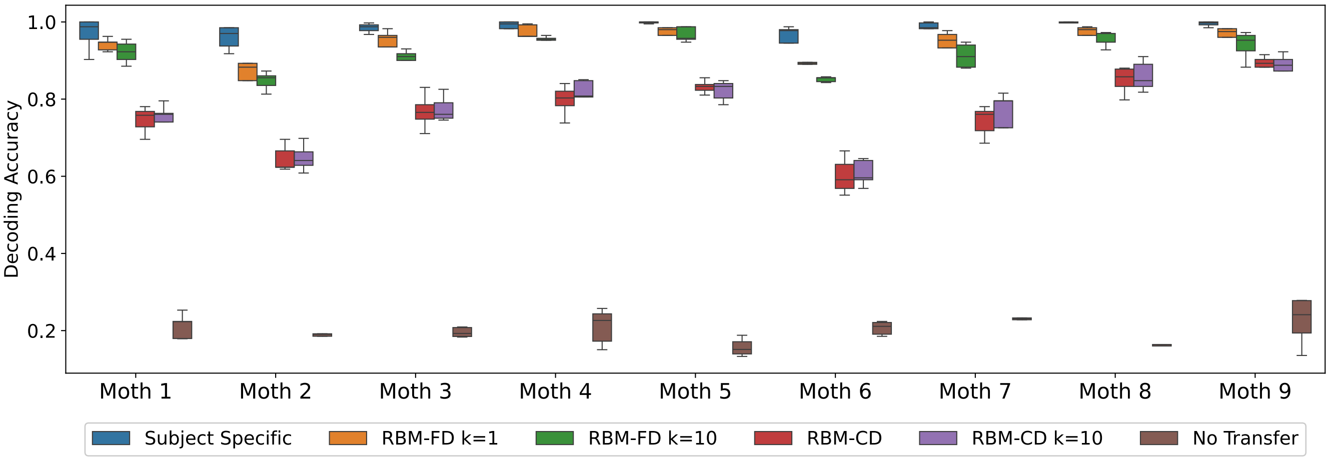

Fig 1–2. Scenario 1 experiment but repeated with k=10.

in Figure 6–1 and 7–1. We note that the cross-validation result is similar to the 100 repeated experiments presented in the main text.

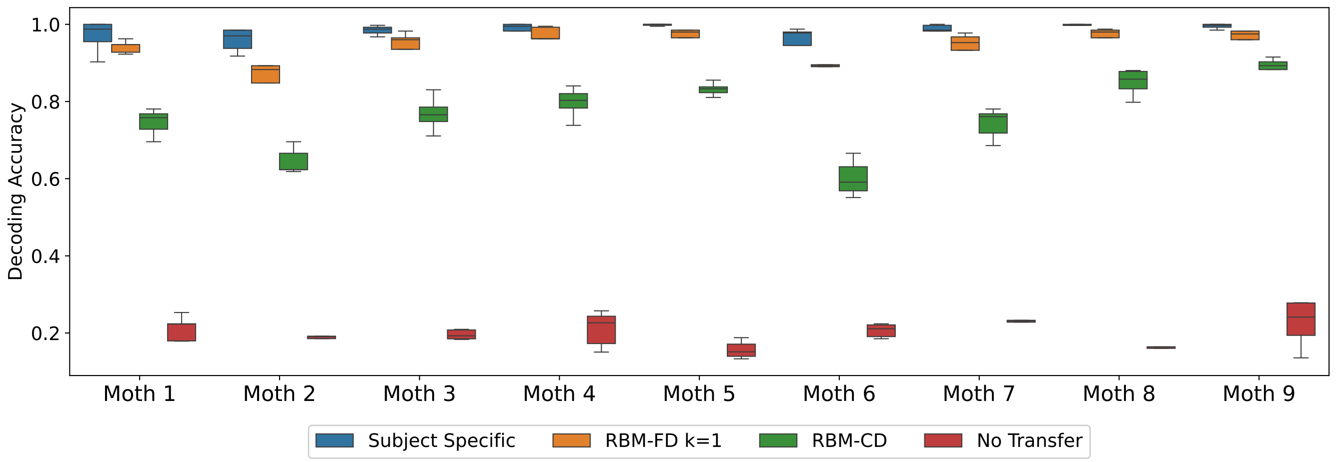

Fig 6–1. Scenario 1 experiment with 95% confidence interval calculated using results from 5-fold cross validation

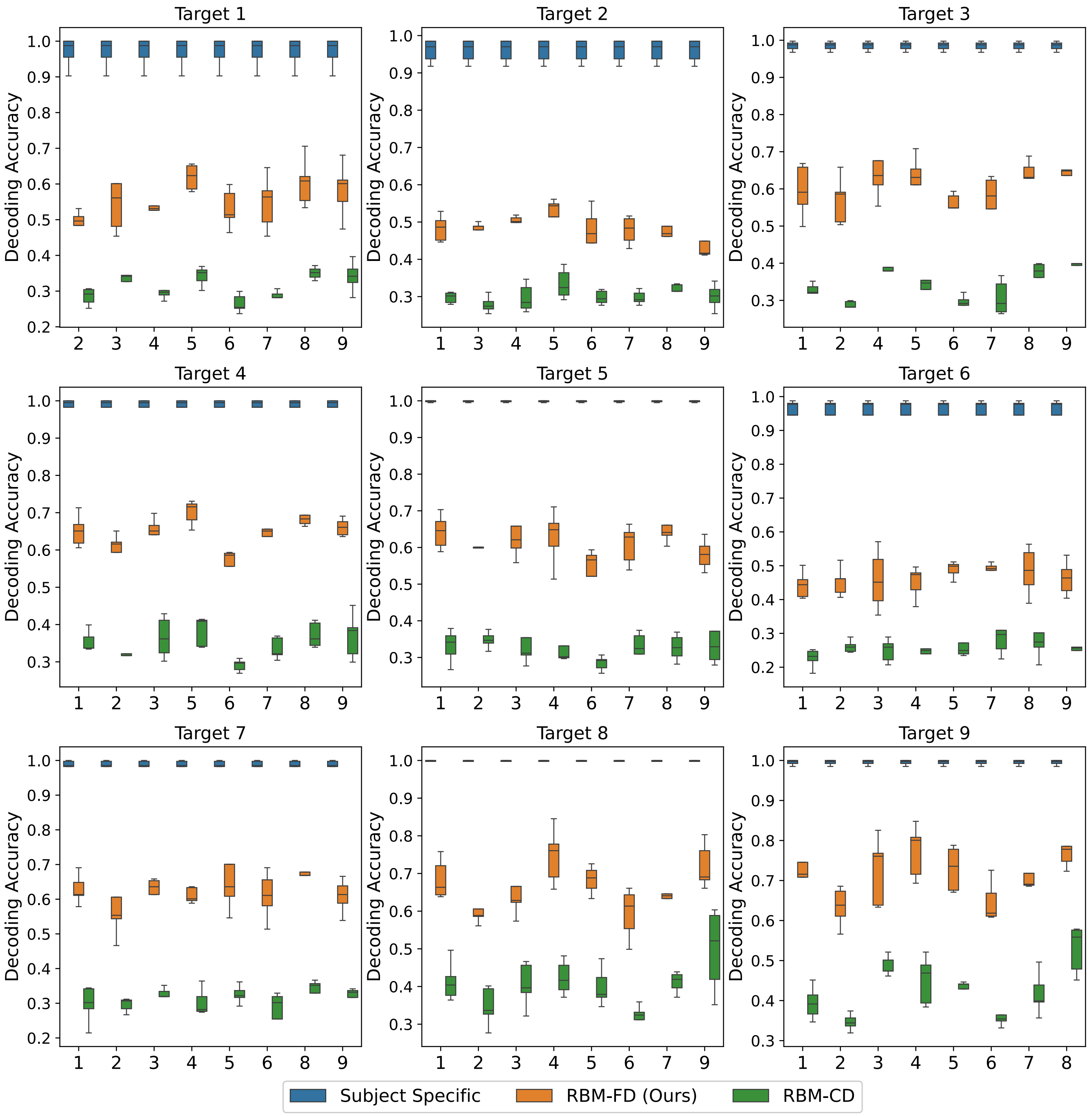

Fig 7–1. Scenario 2 experiment with 95% confidence interval calculated using results from 5-fold cross validation
